# Evidence for proton-pump inhibitor (PPI)-associated dysbiosis in metabolically unhealthy obesity

**DOI:** 10.3389/fendo.2023.1205490

**Published:** 2023-06-15

**Authors:** Melissa A. Burmeister, Tara E. Smith, Timothy K. Fincher, Abby J. Weldon

**Affiliations:** ^1^ William Carey University School of Pharmacy, Department of Pharmaceutical Sciences, Biloxi, MS, United States; ^2^ William Carey University Department of Pharmacy Practice, Biloxi, MS, United States

**Keywords:** proton-pump inhibitor (PPI), metabolically unhealthy obesity (MUO), dysbiosis, inflammation, butyrate, short-chain fatty acids (SCFAs), probiotics

## Abstract

Obesity adversely impacts millions of American adults by predisposing them to significant health risks and further complications. Obesity is differentiated into two groups: metabolically healthy and metabolically unhealthy. In contrast to metabolically healthy counterparts, obese individuals who are metabolically unhealthy display hallmark symptoms of metabolic syndrome (*e.g.*, hypertension, dyslipidemia, hyperglycemia, abdominal obesity). Gastroesophageal reflux disease (GERD) commonly occurs in all obese populations, as do poor dietary habits. Proton-pump inhibitors (PPIs), due to their wide availability, are most often used to treat GERD-related heartburn and other symptoms. Here, we review the evidence on how poor diet as well as short- and long-term use of PPIs adversely affect the gastrointestinal microbiota to cause dysbiosis. Key components of dysbiosis-induced metabolically unhealthy obesity (MUO) associated with PPI use include “leaky gut,” systemic low-grade inflammation, and reduced amounts of short-chain fatty acids (SCFAs) such as butyrate that promote metabolic health. The benefit of using probiotics to mitigate PPI-induced dysbiosis and MUO is also discussed.

## Introduction

Obesity is a chronic, progressive disease with significant adverse health effects and is clinically defined by a body mass index (BMI) >30 kg/m^2^ (1). According to the 2022 National Health and Nutrition Examination Survey, the obesity rate in American adults is 42% (2). Obesity is a significant risk factor for a myriad of comorbidities including type 2 diabetes mellitus (T2DM), cardiovascular disease, metabolic syndrome, gastrointestinal (GI) tract diseases, kidney damage, liver dysfunction, mental illness, and several cancers. Obesity imparts a significant healthcare burden. Healthcare costs are estimated at $172 billion, with heightened costs in severely obese individuals (BMI >35) that increase with age (3).

While most obese individuals exhibit one or more additional metabolic complications, some lack any overt sign of coinciding disease. To differentiate between these two conditions, the medical community coined the terms metabolically unhealthy obesity (MUO) and metabolically healthy obesity (MHO) (4, 5). Obesity is oftentimes accompanied by gastroesophageal reflux disease (GERD), prompting the use of proton-pump inhibitors (PPIs), among other medications, to manage acid reflux and related symptoms (6–8). Mounting evidence indicate that several oral medications including antibiotics and PPIs unfavorably alter the gut microbiota; the resultant dysbiosis is implicated in the etiology and pathogenesis of obesity. Many findings about diet composition, obesity, and PPI use come from preclinical research in animals. Here, we explore the relationships between poor diet, GERD, PPI use, metabolic disease, immune dysfunction, and dysbiosis as well as their associative and potentially causal roles in MUO.

### Metabolically healthy obesity (MHO) vs. metabolically unhealthy obesity (MUO)

MHO is clinical obesity without any comorbidities associated with metabolic syndrome. MHO is characterized by preserved insulin sensitivity, reduced systemic inflammation, less visceral fat, and more favorable hepatic function than MUO counterparts (5, 9). The following MHO criteria are proposed: fasting triglycerides ≤150 mg/dL; high density lipoprotein serum concentration >40 mg/dL in men or >50 mg/dL in women; systolic blood pressure <130 mmHg; diastolic blood pressure <85 mmHg; and fasting blood glucose <100 mg/dL (4, 10). Since MHO individuals have no cardiometabolic disorder, medications for dyslipidemia, hypertension, or diabetes are not required (4, 10). Lack of concrete MHO criteria has led to a large degree of heterogeneity amongst research participants, generating debate about whether to classify MHO as a distinct phenotype or place it on a spectrum that incorporates a devolution to MUO (4, 5). Factors promoting MHO status include healthy diet; regular physical activity; genetic predisposition towards more subcutaneous (vs. visceral) fat; and gut microbiome diversity (5, 10). Metabolic heterogeneity amongst obese individuals is partly governed by differences in adipose tissue physiology, whereby genetic determinants of body fat distribution, depot-specific fat metabolism, adipose tissue plasticity, and adipogenesis predispose some individuals to adiposopathy and MUO (5). Adverse changes in body weight, body composition (*i.e.*, lean vs. fat mass), metabolism (*i.e.*, food intake, energy expenditure (EE), glucose clearance, glucose-stimulated insulin secretion), and fecal microbiota richness are observed in mice fed a high calorie diet and treated with the PPI omeprazole; results varied depending on sex and genetic background (11).

### Gastroesophageal reflux disease (GERD) and proton-pump inhibitors (PPIs)

Individuals with MHO or MUO are equally susceptible to developing GERD (12). Obese individuals who experience GERD commonly use PPIs to relieve heartburn and other discomfort (*e.g.*, chest or upper abdominal pain, dysphagia, globus sensation, food regurgitation) caused by acid reflux. PPIs reduce stomach acid production by inhibiting the H^+^,K^+^-ATPase, an ion pump located on the luminal surface of gastric parietal cells, and blocking hydrochloric acid secretion (13). Through irreversible inhibition of the proton pump, PPIs yield greater acid suppression and have a longer duration of action than other acid-controlling medications such as histamine-2 receptor antagonists or antacids (13). Thus, PPIs are more favorable for reducing gastric acid secretion and relieving pain. PPIs are the medication of choice not only for GERD but also peptic ulcer disease and associated bleeding, *Helicobacter pylori* infection (in combination with antibiotics), NSAID-induced ulcers, erosive esophagitis, Zollinger-Ellison syndrome, and functional dyspepsia (14).

Fueled by over-the-counter availability, PPI usage has steadily increased since 2003, when omeprazole (Prilosec) was FDA-approved for purchase without a prescription (15). Approximately 15 million Americans use PPIs annually (16). The number of documented indications for PPI use has also increased (17). PPIs are commonly administered in the outpatient, ambulatory care setting for GERD-related symptoms and in the inpatient, critical care setting for stress ulcer prophylaxis. Shortly following OTC availability, many PPI users continued to take these medications, even without documented GI complaints and/or diagnoses or other indications for use (17). Individuals still frequently remain on PPIs long-term (clinically defined as >8 weeks) after either being initiated on therapy in non-outpatient settings or self-prescribing (17, 18). The long-term use of PPIs is especially concerning due to numerous possible adverse side effects, including T2DM, dysbiosis, *Clostridium difficile infection* (CDI)-associated diarrhea, enteric infections, increased risk of community-acquired pneumonia, magnesium and vitamin B_12_ deficiency, osteoporosis, bone fractures, and dementia (14, 19–22).

### Dysbiosis and metabolic disease

Gut microbiota are a core participant in host metabolic health by modulating digestion and absorption, whereby foodstuffs are converted into essential nutrients and minerals. A diet enriched in prebiotic and probiotic foods including plant-derived protein while limited in processed foods and animal-derived protein, healthy lifestyle, and environmental and genetic factors all support a diverse and optimal gut microbiota (23–25). The healthy human microbiota exhibits a balance of the phyla Firmicutes and Bacteroidetes, which represent 90% of gut microbiota (26, 27). The remaining dominant phyla include Actinobacteria, Proteobacteria, Fusobacteria, and Verrucomicrobia. A rich microbiota contributes to health by facilitating drug metabolism, synthesis of essential vitamins B and K, and physical and chemical protection against colonization by pathogens (7, 23, 26). These microbiota also ferment fiber and other indigestible polysaccharides, yielding short-chain fatty acids (SCFAs) that beneficially impact body weight, inflammatory status, insulin sensitivity, and glucose and lipid homeostasis (28).

Reduced biodiversity of gut microbiota, coupled with subsequent expansion of disease-promoting pathogens, is referred to as dysbiosis (23). Dysbiosis is a hallmark of inflammatory bowel disease (IBD) and is also associated with several autoimmune, neurological, and metabolic disorders, with causal evidence emerging (23, 29–35). Variations in the composition and abundance of oral and/or gut microbiota, especially at the phylum level, are implicated in metabolic disease (7, 9, 36, 37). Namely, an increase in the Firmicutes to Bacteroidetes (F/B) ratio occurs in overweight and obese individuals (38). High fat diet (HFD)-fed mice show an increase in Firmicutes and decrease in Bacteroidetes proportions, leading to a higher F/B ratio vs. lean mice (36). In obese, human, metabolic syndrome recipients, allogenic fecal microbiota transfer (FMT) using metabolic syndrome donors (vs. post-gastric bypass donors) decreases insulin sensitivity, suggesting that dysbiosis can trigger MUO (39). Conversely, FMT using normal diet-fed and exercised donor mice improves metabolism and inflammatory status in HFD-fed recipients (40). However, FMT using healthy lean donors fails to potentiate the improved insulin sensitivity imparted by consumption of a healthy diet in MUO individuals (41, 42).

The F/B ratio’s validity as a reliable biomarker has been challenged by various confounding factors in study populations and lack of clear correlation between its numerical value and BMI. This discrepancy suggests that dysbiotic gut events impacting metabolic health are more nuanced (9, 27, 43, 44). Compared to MHO individuals, intestinal levels of inflammatory-associated microbiota are elevated in MUO, accompanied by lower bacterial diversity and reduced potential for butyrate production (45–47). Alpha diversity, an index of taxa richness and abundance, is lower in MUO vs. MHO adults and children (9, 47). The genera *Oscillospira* and *Clostridum*, microbial sources of beneficial SCFAs, are more abundant in MHO individuals (9). Butyrate, a key SCFA, exhibits anti-inflammatory properties by reducing pro-inflammatory cytokines and GI mucosal permeability, thereby preventing inflammation mediated by the bacterial endotoxin lipopolysaccharide (LPS) (9, 28). Butyrate-producing bacteria are significantly decreased in T2DM, suggesting that this SCFA confers protection against the development of insulin resistance (9). Family members of Firmicutes and Actinobacteria associated with beneficial metabolic effects are also more abundant in MHO vs. MUO individuals (48).

In contrast, Fusobacteria is more abundant in MUO individuals (9). Despite increased abundance in T2DM individuals, elevated Fusobacteria levels do not significantly correlate with increased BMI (49). Differing from most other microorganisms, Fusobacteria is abundant with intestinal inflammation (9). Fusobacteria are established oral pathogens well-implicated in colorectal cancer, where they upregulate the pro-inflammatory cytokines tumor necrosis factor alpha, interleukin-6, and interleukin-8 as well as cyclooxygenase-2 enzyme (50). As gram-negative microorganisms, Fusobacteria also contribute to inflammation via the LPS component of their cell wall (51). In addition to increased LPS release, elevated cytotoxic reactive oxygen species (ROS) levels, reduced bioavailability of nitric oxide (a central regulator of energy metabolism and body composition), and decreased SCFA production occur with obesity (26, 27, 43). These events create conditions that promote inflammation, induce endothelial dysfunction, and reduce insulin sensitivity, which leads to further inflammation, dyslipidemia, hyperglycemia, and other cardiometabolic dysfunction.

### Diet- and oral PPI-induced dysbiosis

Diet composition and oral ingestion of medications substantially influence microbiota diversity (23, 26, 27, 52). Diets enriched in saturated fat, protein, and complex carbohydrates decrease gut microbiota biodiversity through the production of toxic metabolites or by overfeeding certain families of potentially pathogenic organisms (23). These diets increase gram-negative bacteria like *Escherichia coli* that harbor LPS and decrease the prevalence of favorable gram-positive bacteria that help maintain the gut mucosal barrier to protect against endotoxins (53, 54). Metabolic endotoxemia, approximately a two-fold increase in circulating LPS levels from baseline, is one mechanism by which dysbiosis and leaky gut elicit the systemic inflammation and insulin resistance that characterize MUO (55, 56). Systemic administration of LPS to lean mice increases fat deposition, systemic and tissue-specific inflammation, and insulin resistance to a similar extent as that caused by diet-induced obesity (DIO) (55). Furthermore, serum LPS levels are 1.5-fold greater in obese mice fed a normal chow diet than in lean mice fed a HFD (55). LPS binds with LPS-binding protein (LBP) to trigger the toll-like receptor 4 signaling cascade, which activates the inflammatory immune response (56). Both LPS and LBP are elevated in individuals with obesity or T2DM compared to healthy controls (56). Poor diet is a major culprit in the etiology and pathogenesis of obesity partly through an LPS-mediated mechanism and is linked to GERD, driving PPI use. Obesogenic diets, particularly those high in fat, increase GERD risk by lowering esophageal sphincter (LES) tone, increasing transient LES relaxation, and delaying gastric emptying (57, 58). These diets also elevate intestinal amounts of LPS-releasing, gram-negative bacteria that promote the pro-inflammatory state implicated in abnormal LES relaxation (59, 60). Esophageal microbiome analyses reveal a skewing towards gram-negative populations in esophagitis and Barrett’s esophagus. This profile is strongly linked to GERD-related pathology through LPS-mediated induction of NO, promoting LES relaxtion (61, 62). Several oral medication classes alter the microbiome. With only short-term use, repeated exposure to antibiotics negatively alters microbiome composition, possibly long-term (23). A positive association between antibiotic exposure and weight gain in children has been reported (63). Compared to other commonly used medications such as statins, antibiotics, antidepressants, and metformin, PPIs impart the greatest and most consistent inter-individual variability in gut microbiota (64–68). PPI use is linked to increased risk of CDI by altering CDI-associated taxa, increasing gastric pH, and delaying gastric emptying (69–72). Comprehensive meta-analyses determined that PPI use increases the risk of developing initial and recurrent CDI by two- and 1.5-fold, respectively (73, 74). Strong evidence for PPI-induced risk prompted an FDA-issued drug safety warning (75). Daily PPI use is recognized as a sole, avoidable, independent risk factor for CDI-associated mortality in a dose-dependent fashion (76).

Although PPIs are not pro-inflammatory *per se*, they induce changes in the gut microbiota that cause inflammation. Intestinal amounts of *Enterococcus*, *Clostridium*, and *Lactobacillus* increase with PPI use, whereas those of *Bacteroides* and *Bifidobacteria* decrease, elevating the F/B ratio (77–80). Compared to pre-treatment values, human participants undergoing 8-week treatment with the PPIs esomeprazole, rabeprazole, or lansoprazole had increased fecal amounts of Firmicutes due to bacterial translocation from the oral, nasal, and throat cavities to the intestine (81). Confoundingly, this study did not control for any change in diet post-GERD relief (81). Once daily administration of esomeprazole for 4 weeks increases the fecal abundance of *Streptococcus* (normally found in the upper GI tract), with trends for increased amounts in the saliva and periodontal pocket also observed (81). *Streptococcus* increases oxidative stress in the GI tract via ROS production (80). Increased *Streptococcus* is also associated with duodenal eosinophil infiltration both after short- and long-term PPI therapy (79). The resultant intestinal inflammation is a key factor in the development of systemic, low-grade inflammation. Omeprazole use also increases the abundance of Fusobacteria and Firmicutes in the gastric mucosa of healthy dogs (82). In rats, long-term administration of lansoprazole reduces microbiota diversity and richness, with reduced abundance of *Clostridium* and members of Actinobacteria and Bacteroidetes (28).

Obesity likely increases the risk of stress, anxiety, and depression, especially when metabolic disturbances are present (83). In line with these findings, increased intestinal permeability stemming from PPI use and dysbiosis of gut microbiota is enhanced during psychological stress (78). In mice subjected to water avoidance stress (WAS), once daily administration of the PPIs rabeprazole, omeprazole, or lansoprazole post-stress session exacerbated WAS-induced increases in intestinal permeability and duodenal mast cell infiltration both *in vivo* and *ex vivo*; these phenomena are transferrable via gut microbiome transplantation (78). Expression of multiple duodenal tight junction adhesion molecules (at both the gene and protein levels) is also decreased with PPI treatment (78). Strengthening the notion that stress plays a causal role in the pathogenesis of obesity, PPIs do not increase intestinal permeability in the absence of stress (78).

### Obesity-related and PPI-induced aberrations in short-chain fatty acid (SCFA) production

The microbiome-gut-brain axis is a bidirectional communication network amongst the central nervous system (CNS), autonomic nervous system (ANS), enteric nervous system (ENS), and hypothalamic pituitary adrenal (HPA) axis that maintains GI and neuronal homeostasis (84). Hypothalamic neurons sense microbiota cell wall components to regulate food intake and EE (85). SCFAs are involved in microbiota-gut-brain interactions as substrates of G protein-coupled receptors (GPCRs) to positively influence host functions such as appetite, glucose homeostasis, EE, immunomodulation, and functional integrity of the GI tract (28, 52, 86, 87).

The most common SCFAs produced by the microbiome are butyrate, propionate, and acetate. Butyrate’s protective effects against obesity are pleiotropic (88). Butyrate regulates body weight by promoting EE and reducing energy intake. It induces mitochondrial function in association with up-regulated expression of genes involved in lipolysis and fatty acid β-oxidation. In brown adipose tissue, it promotes thermogenesis via activation of lysine-specific demethylase and β_3_-adrenergic receptors. Along the gut-brain axis, it inhibits weight gain by promoting satiety and reducing food intake by suppressing the activity of hypothalamic orexigenic neurons. Butyrate’s hypophagic and anorectic effects are mediated by increased levels of glucagon-like peptide 1, glucose-dependent insulinotropic polypeptide, and gut hormone peptide YY, as well as up-regulation of the mu-opioid receptor. In the liver, butyrate upregulates antioxidant systems by promoting β-oxidation and stimulating fibroblast growth factor 21 through activation of peroxisome proliferator-activated receptor α. These hepatic events are accompanied by reduced inflammation, lipid deposition, and cholesterol synthesis. In adipose tissue, it induces leptin production and secretion, promotes β-oxidation, and inhibits inflammation. In the pancreas, it promotes insulin secretion and inhibits glucagon secretion. In the gut, it influences the expression of colonic tight junction proteins to control gut permeability (88).

Decreased SCFA production, particularly butyrate-producing microbes, as a consequence of consuming a Western-style diet is implicated in obesity and other metabolic diseases (88, 89). Conversely, dietary supplementation with acetate, propionate, butyrate, or their admixture inhibits HFD-induced weight gain in mice (36). GPR41 and GPR43 are mammalian GPCRs located in adipose tissue, GI epithelium, and lymphatic tissue that are upregulated by circulating LPS and systemic inflammation (90). HFD intake lowers gene transcript levels of GPR41 and GPR43 in adipose tissue and elevates levels in colon vs. lean mice; SCFA supplementation reverses these effects (36). Long-term administration of lansoprazole to rats reduces intestinal and colonic butyrate concentrations, especially in old age (91). Moreover, the abundance of *Lactobacillus* in the ileum is significantly and positively correlated with butyrate concentration in the duodenum and ascending colon and positively correlated with butyrate levels in the jejunum (91). Of note, SCFAs do not always impart beneficial effects on metabolic health. Some preclinical data indicate that signaling at GPR41 and GPR43 is associated with DIO and inflammatory disease (90). These observations reflect the complex manner through which the microbiome regulates inflammation and metabolism.

## Discussion

Obesity is a multifactorial condition associated with multiple concomitant diseases through a myriad of complex mechanisms. Obesity resides on a spectrum ranging from healthy to unhealthy, whereby adipogenesis and inflammation mediate its comorbidities including dyslipidemia, cardiovascular dysfunction, and insulin resistance. FMT data indicate that MUO may stem from unfavorable alterations in gut microbiota (39–42). This dysbiosis simultaneously inhibits the production of beneficial, health-promoting metabolites (*i.e.*, SCFAs) and promotes the production of pro-inflammatory, harmful ones (*i.e.*, LPS).

Genetic and environmental factors influence the microbiome. Diet composition is one key environmental factor. Oral medications such as antibiotics also negatively alter the microbiome, potentially compromising its natural diversity years after initial exposure. Emerging evidence identifies PPIs as another culprit medication class associated with dysbiosis. In most cases, the intended duration of PPI use is only up to 8 weeks. Alarmingly, long-term PPI use is increasingly common in obese and pediatric populations (92, 93). This could permanently alter microbiome composition, and many associative findings and emerging causal evidence indicate that it deleteriously affects metabolic health long-term. Yet the full impact of short- and long-term PPI use on altering gut microbiome composition and the extent to which dysbiosis contributes to MUO in humans remains largely unknown, as no clinical trials have examined these questions to date.

Attempts to prevent/attenuate negative impacts on metabolic health related to PPI-associated dysbiosis might involve curtailing the following: physician overprescribing, direct-to-consumer advertising, misdiagnosis, self-diagnosis, and treating symptoms rather than the cause(s) of acid reflux. Although data are limited, taking probiotics and eating prebiotic foods rich in antioxidants and dietary fiber appear to be beneficial (92, 94, 95). High fiber diet improves metabolic health and mood in T2DM patients (96). In children, once daily co-administration of probiotics substantially reduced dysbiosis occurrence in response to 12-week, once daily esomeprazole vs. esomeprazole treatment alone from 56.2% to 6.2%, respectively (92). Other studies report mixed findings regarding the beneficial effects of supplementation with *Streptococcus*, *Lactobacillus* and/or *Bifidobacterium* on body weight, BMI, waist circumference, and fat mass (97). A clinical trial analyzing the effects of probiotics to reduce dysbiosis and GI discomfort in adult GERD patients using PPIs long-term is currently underway (98). The benefits of probiotic use outweigh any potential risks. Namely, probiotics prevent and treat antibiotic-associated dysbiosis and diarrhea (99). Probiotic use would likely be equally beneficial for PPI-induced dysbiosis and associated metabolic dysfunction.

## Author contributions

MB, TS, TF, and AW conceptualized and drafted the manuscript. All authors contributed to the article and approved the submitted version.
